# Supplementary material for: Novel Driver Strength Index highlights important cancer genes in TCGA PanCanAtlas patients
Source: PeerJ. 2022 Aug 11;10:e13860. doi: 10.7717/peerj.13860 (PMC9375969; doi:10.7717/peerj.13860)
Supplement: Supplemental Information 2 [file peerj-10-13860-s002.zip › COHORTS/genes plots/cohorts/2021_8_16_14_9_distribution_cohorts_UCS.pdf]

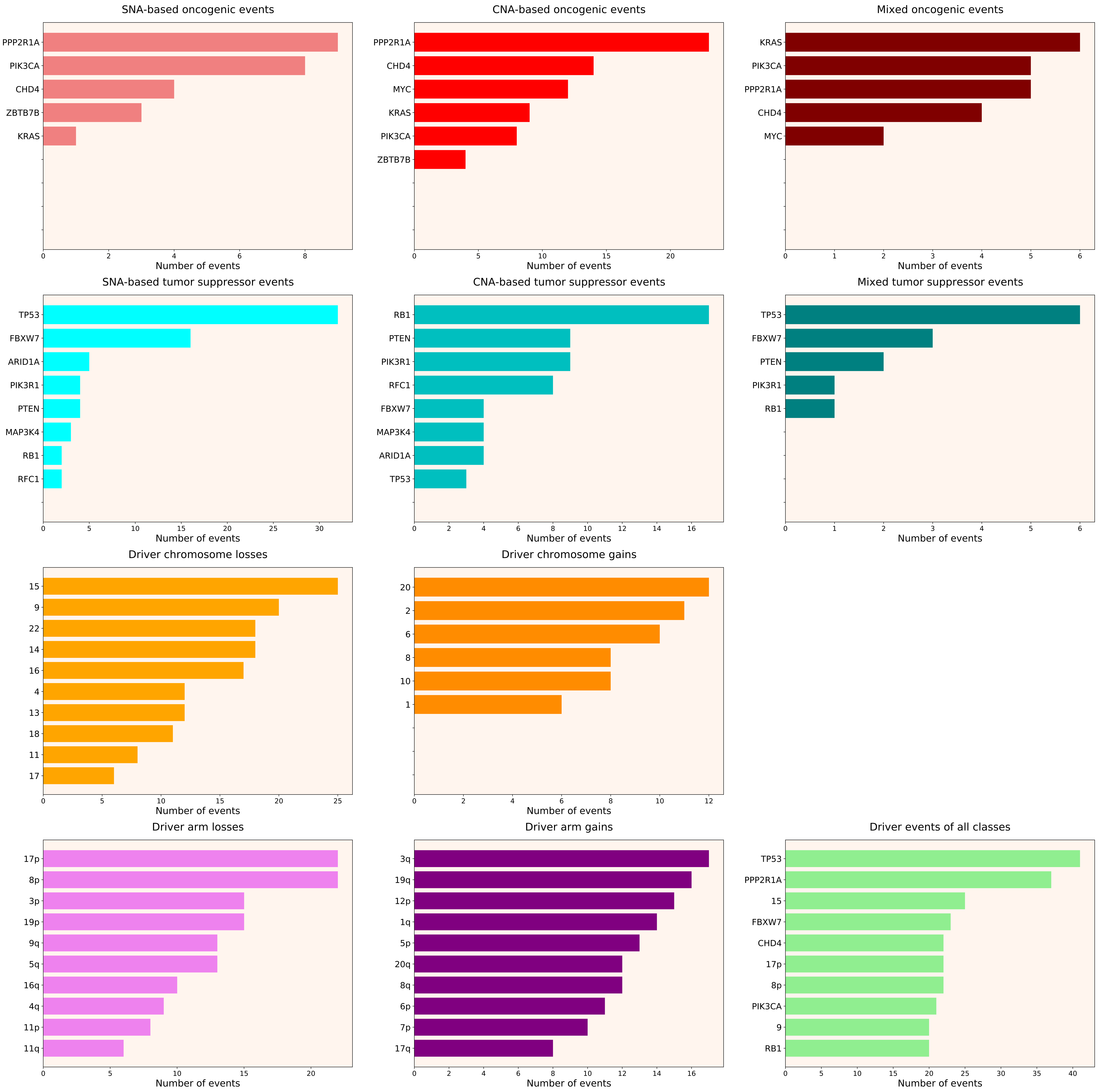
SNA-based oncogenic events
PPP2R1A
PIK3CA
CHD4
ZBTB7B
KRAS
Number of events
CNA-based oncogenic events
PPP2R1A
CHD4
MYC
KRAS
PIK3CA
ZBTB7B
Number of events
Mixed oncogenic events
KRAS
PIK3CA
PPP2R1A
CHD4
MYC
Number of events
SNA-based tumor suppressor events
TP53
FBXW7
ARID1A
PIK3R1
PTEN
MAP3K4
RB1
RFC1
Number of events
CNA-based tumor suppressor events
RB1
PTEN
PIK3R1
RFC1
FBXW7
MAP3K4
ARID1A
TP53
Number of events
Mixed tumor suppressor events
TP53
FBXW7
PTEN
PIK3R1
RB1
Number of events
Driver chromosome losses
15
9
22
14
16
4
13
18
11
17
Number of events
Driver chromosome gains
20
2
6
8
10
1
Number of events
Driver arm losses
17p
8p
3p
19p
9q
5q
16q
4q
11p
11q
Number of events
Driver arm gains
3q
19q
12p
1q
5p
20q
8q
6p
7p
17q
Number of events
Driver events of all classes
TP53
PPP2R1A
15
FBXW7
CHD4
17p
8p
PIK3CA
9
RB1
Number of events
